# Antiplasmodial and Cytotoxic Flavonoids from *Pappea capensis* (Eckl. & Zeyh.) Leaves

**DOI:** 10.3390/molecules26133875

**Published:** 2021-06-25

**Authors:** Nasir Tajuddeen, Tarryn Swart, Heinrich C. Hoppe, Fanie R. van Heerden

**Affiliations:** 1 School of Chemistry and Physics, University of KwaZulu-Natal, Private Bag X01, Scottsville 3209, South Africa; vanheerdenf@ukzn.ac.za; 2 Department of Biochemistry & Microbiology, Rhodes University, Grahamstown 6140, South Africa; g10s2905@campus.ru.ac.za (T.S.); h.hoppe@ru.ac.za (H.C.H.)

**Keywords:** *Pappea capensis*, Sapindaceae, flavonoids, malaria, antiplasmodial, HeLa cells

## Abstract

Ethnobotanical surveys indicate that the Masai and Kikuyu in Kenya, the Venda in South Africa, and the Gumuz people of Ethiopia use *Pappea capensis* for the treatment of malaria. The present study aimed to investigate the phytochemical and antiplasmodial properties of the plant leaves. The bioactive compounds were isolated using chromatographic techniques. The structures were established using NMR, HRMS, and UV spectroscopy. Antiplasmodial activity of *P*. *capensis* leaf extract and isolated compounds against chloroquine-sensitive 3D7 *P. falciparum* was evaluated using the parasite lactate dehydrogenase assay. Cytotoxicity against HeLa (human cervix adenocarcinoma) cells was determined using the resazurin assay. The extract inhibited the viability of *Plasmodium falciparum* by more than 80% at 50 µg/mL, but it was also cytotoxic against HeLa cells at the same concentration. Chromatographic purification of the extract led to the isolation of four flavonoid glycosides and epicatechin. The compounds displayed a similar activity pattern with the extract against *P. falciparum* and HeLa cells. The results from this study suggest that the widespread use of *P. capensis* in traditional medicine for the treatment of malaria might have some merits. However, more selectivity studies are needed to determine whether the leaf extract is cytotoxic against noncancerous cells.

## 1. Introduction

Malaria continues to cause serious morbidity and mortality, especially in Africa, which accounted for over 90% of the approximately 409,000 global malaria deaths in 2019. The problem is complicated by the fact that 67% of the deaths occur in children under the age of five, as well as the high prevalence among pregnant women [1]. In addition, climatic conditions and other factors in sub-Saharan Africa favour malaria transmission [2].

Natural products, whether as mixtures or purified compounds, continue to play a leading role in drug discovery, as drug candidates and as inspiration for the design of novel drugs. This influence of natural products is particularly pronounced in the area of anti-infective agents [3]. Plant natural products have historically produced successful antimalarial drugs. The quinoline antimalarials such as chloroquine and primaquine were inspired by quinine, which is obtained from the barks of South American *Chinchona* plants. Lapachol, a hydroxynaphthquinone with antimalarial activity and found in several plants of the Bignoniaceae, served as the template for the design of atovaquone [4]. Artemisinin, which forms the backbone of the current first-line antimalarial treatments, is isolated from the leaf of the Chinese medicinal herb *Artemisia annua*, or Qinghao. The WHO recommends the use of artemisnin combination therapies, consisting of the rapid acting artemisinin (or its derivatives) and a longer acting partner drug such as an arylamino alcohol or a 4-aminoquinoline, for the treatment of uncomplicated malaria [5]. However, emerging cases of delayed drug response and resistance to artemisinin indicate that the search for new antimalarial agents is an urgent priority.

*Pappea capensis* Eckl. & Zeyh. (Sapindaceae), the only species of the genus *Pappea*, is a small to medium-sized tree with a dense crown found in several types of sub-tropical thickets [6,7]. The tree is endemic to Africa and is mostly found in open woodland, on the edge of riverine areas, on termite mounds, and among rocks. The bark is smooth with a pale grey to brownish colour and the leaves vary in size, with those from arid areas being smaller than those from wetter climates [6,7]. *P. capensis*, also called jacket-plum or wild-plum tree, produces pleasantly flavoured edible fruits, which can be made into a jelly, an alcoholic beverage, or vinegar. The golden yellow seed oil is edible, has a mild purgative effect, and is used topically against baldness and ringworm [6,8]. Zulu traditional healers use the bark and roots for medicinal purposes while unspecified parts of the plant are utilized medicinally for calves [8]. The Ndebele people use an infusion from the leaves for curing painful eyes and the root infusions are given to cattle as an enema or orally for purging [6,8]. The Swahili moisten the root bark with water and use it against chest problems. Masai warriors take a decoction or infusion from the bark as a blood tonic, aphrodisiac and to gain courage. The bark is used for the treatment of venereal diseases and as a protective charm in Botswana, while the leaf is used for nose bleeding [8]. Ethnobotanical surveys show that the Masai of Kenya [9], Gumuz people of Mandura Woreda in Ethiopia [10] and the Kikuyus of Kenya [11] frequently use *P. capensis* to manage malaria. The outcome of an informal interview with Venda people living in Mutale Municipality in Limpopo province indicated that *P. capensis* is effective against malaria and related symptoms [12]. In previous biological studies, the extracts of *P. capensis* twigs and roots showed moderate antiplasmodial activity against the NF54 strain [12,13]. The leaf extract also displayed anti-inflammatory, anti-oxidant, and antimicrobial activities [14,15]. However, despite the widespread use of different parts of *P. capensis* in treating malaria, the antiplasmodial activity of the plant leaves has not been studied previously. Therefore, in search of antiplasmodial compounds from South African medicinal plants, we have investigated the leaves of *P. capensis*.

## 2. Results and Discussion

### 2.1. Chemistry

**Figure 1:**
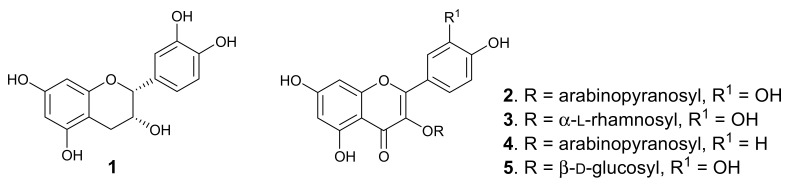
Structures of the isolated compounds from *P. capensis*.

Purification of the dichloromethane-methanol (DCM-MeOH, 1:1) extract of the leaves afforded five known flavonoids, of which four are glycosides. The compounds (Figure 1) were identified, by analysing the spectroscopic data and through comparison with literature values, which showed very close agreement, as epicatechin (**1**) [16], quercetin 3-*O*-arabinopyranoside (guaijaverin, **2**) [17], quercitrin (**3**) [18], kaempferol 3-*O*-arabinopyranoside (juglanin, **4**) [19], and quercetin 3-*O*-β-D-glucoside (isoquercitrin, **5**) [20]. Two major peaks were observed in the HPLC-DAD chromatogram of the leaf extract, corresponding to epicatechin and guaijaverin, while juglanin, quercitrin, and isoquercitrin gave minor peaks. *P*. *capensis* is the only species in the genus *Pappea*, and epicatechin and quercitrin were previously isolated from the plant leaves [15]. However, this is the first report of the isolation of quercetin 3-*O*-arabinopyranoside, kaempferol 3-*O*-arabinopyranoside, and quercetin 3-*O*-β-D-glucoside from the plant leaves. Chemosystematics of the Sapindaceae shows that the family is rich in flavonoids [21,22]. The leaves of plants belonging to the Sapindaceae have been reported to contain flavones, flavonols, including *O*- and *C*-glycosides, and proanthocyanidins [21,23]. In this study, four of the isolated flavonoids from the leaves of *P*. *capensis* are flavonol *O*-glucosides, whereas epicatechin is a monomer of some proanthocyanidin.

### 2.2. Biological Activity

The leaf extract of *P. capensis* and isolated compounds inhibited the viability of *P. falciparum* by more than 80% at 50 µg/mL (Table 1). However, the activity was significantly reduced at a lower concentration of 10 µg/mL. None of the compounds or extract showed 50% parasite inhibition at 10 µg/mL. The extract and compounds were also evaluated for cytotoxicity against HeLa cells. The pattern of antiproliferative activity against HeLa cells was similar to the antiplasmodial activity, suggesting that the activity was not selective. Previous studies have shown that flavonoids and their glycosides have antiplasmodial activity [24]. Houël et al. [25] reported moderate (IC_50_ = 5.5 µM) and weak (IC_50_ = 71.4 µM) activity against FcB1 *P*. *falcirparum* strain for guaijaverin and quercitrin, respectively, without the compounds being cytotoxic to non-cancerous Vero cells. Isoquercitrin was also reported to possess moderate activity against 3D7 (IC_50_ = 4.55 µM) and K1 (IC_50_ = 7.02 µM) strains of *P*. *falciparum* [26]. However, the antiplasmodial activity of juglanin is being reported here for the first time. The discrepancy between our observed results and the reported activity for these compounds might be due to different parasite strains and/or variation in the assay methods.

Common dietary flavonoids, including quercetin and kaempferol, have been reported to inhibit the in vitro viability of 3D7 *P*. *falciparum* [27]. Quercetin has shown a higher antiplasmodial activity than isoquercitrin, suggesting that glycosylation, which makes isoquercetin more polar, reduced the antiplasmodial activity. The superior activity of quercetin might be due to better cell permeability [27]. The compounds investigated in this study are glycosides of quercetin and kaempferol, and their activity was studied in an in vitro model, which may not give an accurate representation of the potency of the compounds, since xenobiotic metabolism of the glycosides, in vivo, may produce more or further less potent aglycones and their conjugates [27], such as sulfate, methyl, and glucuronide derivatives. Additionally, the bioactive compounds in the plant extract might interact synergistically or additively in vivo to give an improved potency, which cannot be observed in the in vitro model used.

Nevertheless, there are conflicting reports of the activity and selectivity of flavonoids against different strains of *P*. *falciparum* in the literature [24,25,26,27,28,29,30], from potent and selective, to weak and unselective. The apparent lack of selectivity in the antiplasmodial activity of flavonoids has been a limiting factor in the further development of this class of compounds as antimalarial agents. Considering the abundance of flavonoids in dietary sources and how easily the flavonoids could be harnessed to combat malaria if the efficacy is proven, a detailed structure–activity relationship study on the antimalarial activity of flavonoids would be worthwhile.

## 3. Materials and Methods

### 3.1. General Procedures

Optical rotations were recorded on a Bellingham and Stanley ADP440+ polarimeter (Bellingham + Stanley Ltd., Longfield Road, Tunbridge Wells, Kent TN2 3EY, UK). NMR spectra were obtained on Bruker AVANCE III (Bruker Corporation, Billerica, MA, USA) spectrometers (400 or 500 MHz for ^1^H and 100 or 125 MHz for ^13^C), using a 5 mm BBOZ probe. The spectra were referenced to residual solvent peaks, δ_H_ 3.31 and δ_C_ 49.03 for CD_3_OD. Mass spectra were recorded on a TOF Waters Micromass LCT Premier mass spectrometer (Waters Corporation, 34 Maple street, Milford, CT, USA) using ESI-ionization in negative or positive modes in MS-grade acetonitrile and methanol solutions. For thin-layer chromatographic analyses, pre-coated TLC silica gel 60 F_254_ (Merck) plates were used. Column chromatography was performed on Merck silica gel (230–400) mesh, and gel filtration chromatography was performed using Sephadex LH-20 (Fluka). Spots on TLC plates were observed under UV light at 254 and 365 nm, and visualised by spraying with *p*-anisaldehyde/H_2_SO_4_ spray reagent (0.5 mL of *p*-anisaldehyde, 10 mL of glacial acetic acid, 4 mL of concentrated H_2_SO_4_ acid and 85 mL of MeOH), followed by heating at 100 °C for 5 min.

### 3.2. HPLC and HPLC Conditions

The HPLC experiment was performed on a Shimadzu LC-20AB Prominence liquid chromatograph (Shimadzu Corporation, 1, Nishinokyo Kuwabara-cho, Nakagyoku, Kyoto, Japan) equipped with a binary pump, an SPD-M20A Prominence diode-array detector, a SIL-20A Prominence autosampler, and a CBM-20A communications bus module. Analytical HPLC analysis was performed using a Phenomenex (00G-4252-B0) Luna column (5 µm, C18 (2), 100 Å, (250 × 4.6 mm)); the flow rate was 0.5 mL/min with isocratic elution at 23 °C and an injection volume of 10 µL. The mobile phase used was 40% methanol-acetonitrile (4:3) containing 0.1% formic acid (solvent B) and H_2_O containing 0.1% formic acid (solvent A). The sample solution (1 mg/mL) was filtered using a PVDF membrane with a pore size of 0.45 µm before injection. Semi-preparative HPLC was performed on a Phenomenex (00G-4252-N0) Luna column (5 µm C18 (2) 100 Å, (250 × 10 mm)), the flow rate was 2.1 mL/min with isocratic elution at 23 °C and an injection volume of 150 µL. The solvents used were 37% methanol-acetonitrile (4:3) containing 0.1% formic acid (solvent B) and H_2_O containing 0.1% formic acid (solvent A). Before injection, the sample solutions (10 mg/mL) were filtered using a PVDF membrane with a pore size of 0.45 µm. HPLC grade acetonitrile and methanol were used, and the detector was set to read wavelengths from 230 to 750 nm.

### 3.3. Plant Material and Preparation of Extract for Bioassay

The leaves of *P. capensis* were collected from the University of KwaZulu-Natal (UKZN) botanical gardens Pietermaritzburg Campus, in March 2019. The leaves were identified by Ms Alison Young, the curator at the University of KwaZulu-Natal (UKZN) botanical gardens. A voucher specimen was prepared and deposited at the Bews herbarium, UKZN School of life Sciences, where it was assigned an accession number (NU0087106). The leaves were air-dried at room temperature in the laboratory and crushed to a coarse powder using a hammer mill. The powdered plant material was weighed and stored in paper bags in a ventilated environment.

For the biological assays, the powdered plant leaves (50 g) were extracted by cold maceration with constant stirring in 500 mL of dichloromethane-methanol (1:1, *v/v*) for 72 h. The extract was filtered and concentrated under reduced pressure using a rotary evaporator to give a green solid mass. A portion of the crude extract (50 mg) was dissolved in minimal amount of methanol, loaded on a polyamide (1.0 g) packed column and eluted three times with methanol (6 mL each). The eluates were combined, evaporated to dryness, weighed, and stored in a refrigerator until needed for the assay.

#### 3.3.1. Isolation of Compounds from the Leaves of *P. capensis*

The powdered leaves of *P. capensis* (950 g) were extracted as described in Section 3.3, but without passing through a polyamide column. From the resulting crude extract, 40.0 g was subjected to VLC using 200 g of silica and 800 mL each of five solvent systems, including hexane-dichloromethane (9:1), dichloromethane-ethyl acetate (20:1), 100% ethyl acetate, ethyl acetate-methanol (5:1), and 100% methanol to give five fractions (A-E). Fractions A (324.8 mg) and B (5.0 g) were composed of fatty material and pigments, respectively, and were ignored. Fraction C (5.2 g) was chromatographed over silica gel packed column and eluted with gradients of dichloromethane-ethyl acetate (8:2, 1:1, 2:8) and ethyl acetate-methanol (8:2) before finally washing the column with 100% methanol, to afford four subfractions (sb-Fr1-4) by pooling together eluates with similar TLC profiles. Sb-fr1-3 were mostly composed of pigments and oils, and were ignored. Silica gel column chromatography of sb-fr4 (1.2 g) by isocratic elution with Hexane-ethyl acetate (1:3) afforded epicatechin (**1**) (128.7 mg) and a yellow residue (550 mg), which was further purified over silica gel column, followed by chromatography over Sephadex LH-20 to afford quercetin 3-*O*-arabinopyranoside (**2**) (33.2 mg), and a yellow residue (120 mg) with one major and two other minor spots on TLC. Attempts to separate the yellow residue using silica gel and Sephadex LH-20 chromatography were not successful. Further semipreparative HPLC purification of the yellow residue by isocratic elution over 60 min using 37% methanol-acetonitrile (4:3, 0.1% formic acid) and 63% H_2_O (0.1% formic acid) at a flow rate of 2.1 mL/min gave quercetin 3-*O*-β-D-glucoside (**5**) (0.9 mg), quercetin 3-*O*-arabinopyranoside (**2**), quercetin 3-*O*-α-L-rhamnoside (**3**) (1.2 mg) and kaempferol 3-*O*-arabinopyranoside (**4**) (0.9 mg). Fractions D (15.15 g) and E (11.8 g) contained a sticky grey solid mass that dissolved only in water, but was insoluble in methanol, ethyl acetate, and chloroform, and so was not further investigated.

##### Spectroscopic Data of Compounds

Epicatechin (**1**): brown powder, [α]_D_^29^ -42.12 (*c* = 1.2, MeOH), ^1^H NMR (500 MHz, CD_3_OD): δ_H_ 6.98 (1H, d, *J* = 2.0 Hz, H-2′), 6.80 (1H, dd, *J* = 8.3, 2.0 Hz, H-6′), 6.76 (1H, d, *J* = 8.3 Hz, H-5′), 5.95 (1H, d, *J* = 2.3 Hz, H-8), 5.93 (1H, d, *J* = 2.3 Hz, H-6), 4.82 (1H, m, H-2), 4.18 (1H, m, H-3), 2.86 (1H, dd, *J* = 16.6, 4.5 Hz, H-4a), 2.74 (1H, dd, *J* = 16.8, 2.8 Hz, H-4b). ^13^C NMR (125 MHz, CD_3_OD): δ_C_ 157.9 (C-5), 157.6 (C-7), 157.3 (C-9), 145.9 (C-3′), 145.7 (C-4′), 132.2 (C-1′), 119.4 (C-6′), 115.9 (C-5′), 115.3 (C-2′), 100.1 (C-10), 96.4 (C-6), 95.9 (C-8), 79.8 (C-2), 67.4 (C-3), 29.2 (C-4). HR-ESI-(-)-MS: *m/z* 289.0705 [M-H]^−^ (Calculated for C_15_H_13_O_6_, 289.0712).

Quercetin 3-*O*-arabinopyranoside (guaijaverin) (**2**): yellow solid, [α]_D_^29^ -54.07 (*c* = 0.5, MeOH), UV (MeOH/ACN): λ_max_ 255, 355 nm; ^1^H NMR (400 MHz, CD_3_OD) δ_H_ 7.74 (1H, d, *J* = 2.1 Hz, H-2′), 7.57 (1H, dd, *J* = 8.5, 2.1 Hz, H-6′), 6.88 (1H, d, *J* = 8.3 Hz, H-5′), 6.39 (1H, d, *J* = 1.9 Hz, H-8), 6.20 (1H, d, *J* = 1.9 Hz, H-6), 5.16 (1H. d, *J* = 6.5 Hz, H-1″), 3.90 (1H, dd, *J* = 8.4, 6.5 Hz, H-2″), 3.81-3.84 (2H, m, H-4″,5″), 3.65 (1H, dd, *J* = 8.4, 3.1 Hz, H-3″), 3.44 (1H, m, H-5″). ^13^C NMR (100 MHz, CD_3_OD) δ_C_ 179.5 (C-4), 166.1 (C-7), 163.0 (C-5), 158.7 (C-2), 158.5 (C-9), 150.0 (C-4′), 146.0 (C-3′), 135.6 (C-3), 123.1 (C-1′), 122.9 (C-6′), 117.5 (C-2′), 116.2 (C-5′), 105.6 (C-10), 104.7 (C-1″), 100.0 (C-6), 94.8 (C-8), 74.1 (C-3″), 72.9 (C-2″), 69.1 (C-4″), 66.9 (C-5″). HPLC R_t_: 25.334 min; HR-ESI-(-)-MS: *m/z* 433.0775 [M-H]^−^ (Calculated for C_20_H_17_O_11_, 433.0771).

Quercetin 3-*O*-α-L-rhamnoside (quercitrin) (**3**): yellow solid, [α]_D_^29^ -136.93 (*c* = 1.1, MeOH), UV (MeOH/ACN): λ_max_ 255, 350 nm; ^1^H NMR (400 MHz, CD_3_OD): δ_H_ 7.34 (1H, d, *J* = 2.0 Hz, H-2′), 7.30 (1H, dd, *J* = 8.5, 2.0 Hz, H-6′), 6.87 (1H, d, *J* = 8.5 Hz, H-5′), 6.36 (1H, d, *J* = 2.0 Hz, H-8), 6.19 (1H, d, *J* = 2.0 Hz, H-6), 5.35 (1H, d, *J* = 1.2 Hz, H-1″), 4.22 (1H, dd, *J* = 3.2, 1.7 Hz, H-2″), 3.75 (1H, dd, *J* = 9.2, 3.2 Hz, H-3″), 3.45 (1H, m, H-5″), 3.34 (1H, d, *J* = 9.4 Hz, H-4″), 0.94 (3H, d, *J* = 6.2 Hz, H-6″). ^13^C NMR (100 MHz, CD_3_OD): δ_C_ 179.6 (C-4), 165.9 (C-7), 163.2 (C-5), 159.3 (C-2), 158.5 (C-9), 149.8 (C-3′), 146.4 (C-4′), 136.2 (C-3), 123.0 (C-1′), 122.9 (C-6′), 117.0 (C-2′), 116.4 (C-5′), 105.9 (C-10), 103.5 (C-1″), 99.8 (C6-), 94.8 (C-8), 73.3 (C-4″), 72.1 (C-3″), 72.0 (C-2″), 71.9 (C-5″), 17.7 (C-6″). HPLC R_t_: 29.504 min; HR-ESI-(-)-MS: *m/z* 447.0915 [M-H]^−^ (Calculated for C_21_H_19_O11, 447.0927).

Kaempferol 3-*O*-arabinopyranoside (juglanin) (**4**): yellow solid, UV (MeOH/ACN): λ_max_ 265, 347 nm; ^1^H NMR (500 MHz, CD_3_OD): δ_H_ 8.06 (2H, d, *J* = 8.0 Hz, H-2′,6′), 6.89 (2H, d, *J* = 8.0 Hz, H-3′,5′), 6.41 (1H, brs, H-8), 6.21 (1H, brs, H-6), 5.13 (1H, d, *J* = 5.6 Hz, H-1″), 3.89 (1H, t, *J* = 6.7, H-2″), 3.79-3.76 (1H, brs, H-4″,5a″), 3.63 (1H, brs, H-3″), 3.40 (1H, d, *J* = 11.2 Hz, H-5b″). ^13^C NMR (125 MHz, CD_3_OD): δ_C_ 179.6 (C-4), 166.2 (C-7), 163.1 (C-5), 161.6 (C-4′), 158.9 (C-2), 158.5 (C-9), 135.6 (C-3), 132.3 (C-2′,6′), 122.7 (C-1′), 116.3 (C-3′,5′), 105.6 (C-10), 104.4 (C-1″), 100.0 (C-6), 94.8 (C-8), 74.0 (C-3″), 72.7 (C-2″), 68.9 (C-4″), 66.7 (C-5″). HPLC R_t_: 34.737 min; HR-ESI-(-)-MS: *m/z* 417.0829 [M-H]^−^ (Calculated for C_20_H_17_O_10_, 417.0822).

Quercetin 3-*O*-β-D-glucoside (isoquercitrin) (**5**): yellow solid, [α]_D_^29^ -32.70 (*c* = 0.18, MeOH), UV (MeOH/ACN): λ_max_ 255, 355 nm; ^1^H NMR (500 MHz, CD_3_OD): δ_H_ 7.70 (1H, d, *J* = 2.1 Hz, H-2′), 7.59 (1H, dd, *J* = 8.5, 2.1 Hz, H-6′), 6.87 (1H, d, *J* = 8.5 Hz, H-5′), 6.4 (1H, brs, H-8), 6.21 (1H, d, *J* = 2.0 Hz, H-6), 5.24 (1H, d, *J* = 7.6 Hz, H-1″), 3.71-3.42 (undefined sugar protons). ^13^C NMR (500 MHz, CD_3_OD): δ_C_ 179.6 (C-4), 166.4 (C-7), 161.6 (C-5), 158.6 (C-2), 158.2 (C-9), 150.4 (C-4′), 145.7 (C-3′), 135.2 (C-3), 122.6 (C-6′), 121.9 (C-1′), 117.4 (C-2′), 116.0 (C-5′), 105.8 (C-10), 105.2 (C-1″), 100.1 (C-6), 94.4 (C-8), 77.7 (C-5″), 75.5 (C-3″), 71.0 (C-2″), 70.0 (C-4″), 62.0 (C-6″). HPLC R_t_: 20.216 min; HR-ESI-(-)-MS: *m/z* 463.0881 [M-H]^−^ (Calculated for C_21_H_19_O_12_, 463.0877).

### 3.4. Antimalarial Assay

#### 3.4.1. The Parasites

Malaria parasites (*Plasmodium falciparum* strain 3D7) were maintained in RPMI 1640 medium containing 2 mM L-glutamine and 25 mM Hepes (Lonza). The medium was further supplemented with 0.5% *w*/*v* Albumax II, 20 mM glucose, 0.65 mM hypoxanthine, 60 µg/mL gentamycin and 2–4% haematocrit human red blood cells. The parasites were cultured at 37 °C under an atmosphere of 5% CO_2_, 5% O_2_, 90% N_2_ in a sealed T75 culture flask [31].

#### 3.4.2. Assessment of In Vitro Antiplasmodial Activity

The extracts and pure compounds were initially screened in duplicate at a single concentration of 50 µg/mL, followed by 10 µg/mL. Compounds showing less than 50% viability without cytotoxicity at 10 µg/mL were screened for an IC_50_ value. Parasite viability was determined by measuring the activity of parasite lactate dehydrogenase (pLDH), as described by Makler et al. [32]. Serial dilutions of the extracts and compounds were added to in vitro cultures of *P. falciparum* (strain 3D7) in 96-well plates (1% haematocrit, 2% parasitaemia). After 48 h of incubation, 20 µL of culture was removed from each well and combined with 125 µL of a mixture of Malstat (0.18 M lactic acid, 0.13 mM 3-acetylpyridine adenine dinucleotide, 0.16% Triton X-100, 44 mM Tris, pH 9) and NBT/PES (0.39 mM nitro blue tetrazolium, 0.05 mM phenazine ethosulfate) solutions in a fresh 96-well plate. The purple product formed, which indicates the presence of pLDH, was quantified in a Spectramax M3 microplate reader (Abs_620_). The Abs_620_ reading in each well is an indication of the pLDH activity, and hence the number of parasites present. The % parasite viability, as indicated by the pLDH activity in treated wells relative to untreated controls, was calculated for each compound/extract. The parasitized RBCs in the absence of test compounds were taken as untreated control with 100% viability. For each compound, percentage viability was plotted against Log of compound concentration and the IC_50_ value (50% inhibitory concentration) was obtained from the resulting dose–response curve by non-linear regression. Chloroquine was used as a positive control drug (IC_50_ values range from 0.01 to 0.05 µM).

### 3.5. In Vitro Cytotoxicity Assay

HeLa (human cervix adenocarcinoma) cells (Cellonex, Johannesburg, South Africa) were plated in 96-well plates at a density of 2 × 10^4^ cells per well in medium consisting of DMEM supplemented with 10% fetal bovine serum and penicillin/streptomycin/amphotericin B antibiotics. After an overnight incubation at 37 °C in a humidified 5% CO_2_ incubator, test samples were added to a final concentration of 50 µg/mL or 10 µg/mL to the cells in triplicate wells, bringing the total medium volume per well to 200 µL, and incubation continued for 24 h. Twenty µL resazurin stock solution (0.6 mM resazurin in phosphate-buffered saline) was added to each well, and, after a 4 h incubation, fluorescence (Exc_560_/Emm_590_) was measured in a Spectramax M3 plate reader. After subtracting background readings obtained from empty wells, the fluorescence values were used to calculate percentage cell viability in treated wells relative to wells containing untreated control cells.

## 4. Conclusions

The leaf extract of *P. capensis* displayed antiplasmodial activity against *P. falciparum* at a concentration at which it was also cytotoxic to cancerous HeLa cells. Investigation of the extract afforded five compounds, of which three flavonoid glycosides were identified for the first time in the plant leaves. The glycosides showed similar activity pattern as the extract. However, the reported use of *P. capensis* leaves in traditional medicine suggests that there might be some other compounds present in the extract that act to mitigate the cytotoxicity.

## Figures and Tables

**Table 1:** In vitro antiplasmodial and cytotoxic activity of the extract and isolated compounds. Viability of *P*. *falciparum* was assessed after 48 h of incubation with the extract/compound, while HeLa cells’ viability was determined after 24 h incubation.

Compound	Viability% ± SD (50 µg/mL)		Viability% ± SD (10 µg/mL)	
	3D7	HeLa	3D7	HeLa
*P. capensis*	12.1 ± 0.2	1.9 ± 0.2	93.1 ± 3.8	71.4 ± 6.1
**2**	19.1 ± 0.5	2.8 ± 0.1	68.4 ± 3.4	72.4 ± 5.4
**3**	16.2 ± 2.2	1.4 ± 0.1	66.8 ± 2.6	71.3 ± 4.5
**4**	18.1 ± 1.0	3.1 ± 0.1	83.1 ± 3.5	70.9 ± 3.7
**5**	18.4 ± 2.9	1.9 ± 0.6	58.7 ± 1.9	64.8 ± 2.2
Chloroquine ^a^	-	-	<10 ± 2.1	-
Emetine ^b^	-	-	-	<5 ± 1.3

^a^ IC_50_ against *Plasmodium falciparum* 3D7 strain 0.0045 µg/mL (0.014 μM), ^b^ IC_50_ against HeLa cells IC_50_ = 0.192 µg/mL (0.04 µM).

## Data Availability

Not applicable.

## Sample Availability

Samples of the tested compounds are available.
